# Biophysical validation of serotonin 5-HT_2A_ and 5-HT_2C_ receptor interaction

**DOI:** 10.1371/journal.pone.0203137

**Published:** 2018-08-29

**Authors:** Daniel E. Felsing, Noelle C. Anastasio, Joanna M. Miszkiel, Scott R. Gilbertson, John A. Allen, Kathryn A. Cunningham

**Affiliations:** 1 Center for Addiction Research, University of Texas Medical Branch, Galveston, Texas, United States of America; 2 Department of Pharmacology and Toxicology, University of Texas Medical Branch, Galveston, Texas, United States of America; 3 Department of Chemistry, University of Houston, Houston, Texas, United States of America; Universite de Rouen, FRANCE

## Abstract

The serotonin (5-HT) 5-HT_2A_ receptor (5-HT_2A_R) and 5-HT_2C_ receptor (5-HT_2C_R) in the central nervous system are implicated in a range of normal behaviors (e.g., appetite, sleep) and physiological functions (e.g., endocrine secretion) while dysfunctional 5-HT_2A_R and/or 5-HT_2C_R are implicated in neuropsychiatric disorders (e.g., addiction, obesity, schizophrenia). Preclinical studies suggest that the 5-HT_2A_R and 5-HT_2C_R may act in concert to regulate the neural bases for behavior. Here, we utilize three distinct biophysical and immunocytochemistry-based approaches to identify and study this receptor complex in cultured cells. Employing a split luciferase complementation assay (LCA), we demonstrated that formation of the 5-HT_2A_R:5-HT_2C_R complex exists within 50 nm, increases proportionally to the 5-HT_2C_R:5-HT_2A_R protein expression ratio, and is specific to the receptor interaction and not due to random complementation of the luciferase fragments. Using a proximity ligation assay (PLA), we found that cells stably expressing both the 5-HT_2A_R and 5-HT_2C_R exhibit 5-HT_2A_R:5-HT_2C_R heteroreceptor complexes within 40 nm of each other. Lastly, bioluminescence resonance energy transfer (BRET) analyses indicates the formation of a specific and saturable 5-HT_2A_R:5-HT_2C_R interaction, suggesting that the 5-HT_2A_R and 5-HT_2C_R form a close interaction within 10 nm of each other in intact live cells. The bioengineered receptors generated for the LCA and the BRET exhibit 5-HT-mediated intracellular calcium signaling as seen for the native receptors. Taken together, this study validates a very close 5-HT_2A_R:5-HT_2C_R interaction in cultured cells.

## Introduction

The metabotropic serotonin (5-HT) 5-HT_2_ receptor (5-HT_2_R) family consists of three isoforms (5-HT_2A_R, 5-HT_2B_R, 5-HT_2C_R) that share ~50% total sequence homology and ~80% sequence homology within their seven transmembrane domains [1]. The 5-HT_2A_R and 5-HT_2C_R localized within the central nervous system are implicated in a range of normal behaviors (e.g., appetite, sleep) and physiological functions (e.g., endocrine secretion) while dysfunctional brain 5-HT_2A_R and/or 5-HT_2C_R are implicated in neuropsychiatric disorders (e.g., addiction, obesity, schizophrenia). Antagonism of the 5-HT_2A_R is a common feature of atypical antipsychotics employed in schizophrenia [2] and is the mode of action for the recently FDA-approved pimavanserin in the treatment of psychosis in Parkinson’s disease [3]. Furthermore, the 5-HT_2C_R agonist lorcaserin is FDA-approved for the treatment of obesity [4]. Biochemical, behavioral and pharmacological studies indicate that the 5-HT_2A_R and 5-HT_2C_R interact in rodents *in vivo* [5–10] raising the possibility that the 5-HT_2A_R and 5-HT_2C_R may act in concert to regulate the neural bases for behavior (for reviews) [11, 12].

The 5-HT_2A_R and 5-HT_2C_R transcripts co-exist in brain regions particularly associated with the limbic-corticostriatal circuitry [13–16], while the protein for these receptors co-localizes to the same neurons in the rat medial prefrontal cortex (mPFC) [17]. We recently demonstrated that the two receptors form a protein complex in the mPFC assessed by co-immunoprecipitation [5]. Expression of this complex was associated with phenotypic levels of impulsivity in the rat [5], suggesting the behavioral significance of a 5-HT_2A_R:5-HT_2C_R protein complex. Based upon reported evidence of a 5-HT_2A_R and 5-HT_2C_R association [5–10], we conducted biophysical studies to further define and validate the interaction between these homologous receptors at the single cell level.

The 5-HT_2A_R and the 5-HT_2C_R are G protein-coupled receptors (GPCRs) that interact with Gα_q/11_ to activate the enzyme phospholipase Cβ which generates intracellular second messengers inositol-1,4,5-trisphosphate and diacylglycerol, leading to increased calcium release from intracellular stores (Ca_*i*_^2+^) [18, 19]. The main functional GPCR unit for the 5-HT_2A_R [20, 21] and 5-HT_2C_R [22–25] is proposed to be their homomeric form. There is also evidence that 5-HT_2A_R can form protein complexes with other GPCRs, including the metabotropic glutamate mGlu_2_ receptor (mGlu_2_R) [26], dopamine D_2_ receptor (D_2_R) [27], cannabinoid CB_1_ receptor (CB_1_R) [28], and 5-HT_1A_ receptor (5-HT_1A_R) [29]. Likewise, protein:protein interactions with the 5-HT_2C_R have been reported with the ghrelin receptor (growth hormone secretagogue receptor 1α, GHS1αR) [30], the melatonin MT_2_ receptor (MT_2_R) [31], and the N-methyl-D-aspartate (NMDA)-gated ion channel subunit GluN2A [32]. The detection of the 5-HT_2A_R:5-HT_2C_R protein complex in cellular models [8] and rodent brain [5, 17] led us to develop biophysical approaches to further define this interaction using *in vitro* cellular models. In the present study, we tested if a 5-HT_2A_R:5-HT_2C_R interaction occurs in cultured cells using three complementary biophysical techniques with increasing spatial resolution. Our findings indicate these receptors form a close biophysical interaction within 10 nm in living cells and provide validation [8] and novel insights into the 5-HT_2A_R:5-HT_2C_R heteromeric receptor interaction.

## Materials and methods

### Compounds and ^3^H-radioligands

All purchased compounds were >98% pure according to the manufacturers. Coelentrazine H (Thermo Scientific, Waltham, MA) was dissolved in ethanol as a stock solution (5 mM). Serotonin (5-HT) hydrochloride (Acros Organics, ThermoFischer Scientific, Pittsburgh, PA), d-luciferin (Gold Biotechnology, St. Louis, MO), and mianserin hydrochloride (Sigma Aldrich, St. Louis, MO) were dissolved in dimethyl sulfoxide (DMSO) (10 mM) and prepared fresh daily. [^3^H]-Mesulergine (84.7 Ci/mmol) and [^3^H]-ketanserin (47.3 Ci/mmol) were purchased from PerkinElmer Life Sciences (Waltham, MA).

### DNA and plasmid constructs

The cDNAs encoding human 5-HT_2A_R and 5-HT_2C-INI_R (non-edited isoform) in pcDNA3.1+ vector were obtained from UMR cDNA Resource Center (Rolla, MO) and employed in the Ca_*i*_^2+^ release assay and luciferase complementation assay (LCA). Split luciferase constructs for the LCA were created using template mammalian expression vectors and standard cloning techniques: the N-terminus of the human rapamycin-binding domain of mammalian target of rapamycin (FRB) fused to the N-terminus of luciferase (NLuc) (pcDNA3.1-V5_HIS TOPO; FRB-NLuc) and the N-terminus of human FK506-binding protein 12 (FKBP) fused to the C-terminus of luciferase (CLuc) (pEF6-V5_HIS TOPO; FKBP-CLuc) were obtained from Dr. David Piwinca-Worms [33, 34]. Briefly, the FRB was replaced with 5-HT_2C-_R by PCR amplification of the open reading frame of the 5-HT_2C_R followed by ligation into the FRB-NLuc fusion vector to create the 5-HT_2C_R-NLuc plasmid. The FKBP was similarly replaced with 5-HT_2A_R in the FKBP-CLuc fusion vector to create the 5-HT_2A_R-CLuc plasmid. The resultant plasmids expressed 5-HT_2C_R-NLuc or 5-HT_2A_R-CLuc (verified by Western blot; data not shown). Because the N-terminus of the 5-HT_2A_R and 5-HT_2C_R is located extracellularly, this attachment of the NLuc (or CLuc) to the C-terminus of either receptor results in intracellular expression of the luciferase fragments. Bioluminescence resonance energy transfer (BRET) plasmids were created and synthesized by Genscript (Piscataway, NJ) for the 5-HT_2A_R, 5-HT_2C_R and the β_2_-adrenergic receptor (β_2_-AR, control for specificity of the BRET interaction). The 5-HT_2A_R and β_2_-AR gene sequences were codon optimized, fused with an N-terminus signal hemagglutinin (HA) sequence, and a C-terminus *Renilla* luciferase (RLuc) sequence in the open reading frame [35]; the 5-HT_2A_R-RLuc and β_2_-AR-RLuc served as the donor constructs. The 5-HT_2C_R gene sequence was fused with a C-terminus enhanced yellow fluorescent (eYFP) protein sequence in the open reading frame [35]; the 5-HT_2C_R-eYFP served as the acceptor construct. All three BRET constructs were subcloned into pcDNA 3.1+ using 5’ BamH1 and 3’ Xba1 restriction sites. The coding regions of all plasmids were entirely sequenced and verified prior to use (Molecular Genomics Core, University of Texas Medical Branch, Galveston, TX).

### Cell culture and transfection

Human embryonic kidney 293 cells (HEK293; CRL-1573™, ATCC, Washington, DC) were cultured as a monolayer in Dulbecco’s modified Eagle’s medium (DMEM) (Gibco, Invitrogen, Waltham, MA) supplemented with 10% fetal bovine serum (FBS) (Gibco, Invitrogen, Waltham, MA) and 1% penicillin/streptomycin (Gibco, Invitrogen, Waltham, MA) in 6-well plates (Thermo Scientific, Waltham, MA). Cells were incubated at 37°C in a 5% CO_2_ and 85% relative humidity. HEK293 cells from passage 7 to 18 (passage one was defined as the first plate of cells from our liquid nitrogen stock) were transiently transfected at 80% confluency in a 6-well plate with 10 μL of Lipofectamine 2000 (Invitrogen, Waltham, MA; according to manufacturer’s protocol) in antibiotic-free Opti-MEM (Gibco, Invitrogen, Waltham, MA) supplemented with 5% dialyzed FBS (Gibco, Invitrogen, Waltham, MA) overnight. For luciferase complementation assays (LCA), varying ratios of 5-HT_2C_R-NLuc:5-HT_2A_R-CLuc plasmids (1:2, 1:1, 2:1, 3:1) with a total of 1 μg cDNA/well were transiently transfected into HEK293 cells. Similarly, 1 μg total cDNA/well was used to transfect cells for analyses of 5-HT_2A_R and 5-HT_2C_R signaling through intracellular calcium release (Ca_*i*_^2+^) assays. For BRET assays, 100 ng of donor plasmid (5-HT_2A_R-RLuc or β_2_AR-Rluc) and increasing amounts (1–20 fold) of acceptor plasmid (5-HT_2C_R-eYFP) were added to each well of a 6-well plate.

We generated a dual stably-transfected cell line by transfecting 5-HT_2A_R-CHO cells with a 5-HT_2C_R construct (5-HT_2A+2C_R-CHO cells) which was employed in the proximity ligation assay (PLA). A Chinese hamster ovary cell line (CHO-K1) stably transfected with the 5-HT_2A_R (5-HT_2A_R-CHO; FA4 cells) [36–38] was a generous gift of Drs. Kelly A. Berg and William P. Clarke (University of Texas Health Science Center at San Antonio, San Antonio, TX). This line expresses the transfected 5-HT_2A_R in the p198-DHFR plasmid containing a hygromycin resistance gene. The parental CHO cell line did not express detectable amounts of any 5-HT_2_R mRNAs [39]. The 5-HT_2A_R-CHO cells were stably transfected with the 5-HT_2C_R in pcDNA3.1+ containing a G418 resistance gene to generate the dual-expressing 5-HT_2A+2C_R-CHO clonal cell line (“FA4E4”). Stably transfected 5-HT_2A+2C_R-CHO cells from passage 8 to 15 (passage 1 was defined as the first plate of cells stably transfected with both 5-HT_2A+2C_R) were cultured at 37°C, 5% CO_2_, and 85% relative humidity in GlutaMax α-MEM (Invitrogen, Carlsbad, CA), 5% fetal bovine serum (Atlanta Biologicals, Atlanta, GA), 100 μg/mL hygromycin (Mediatech, Manassas, VA) and G418 (Corning, Manassas, VA) and were passaged when they reached 70–80% confluency.

### Intracellular calcium (Ca_*i*_^2+^) release assay

Serotonin-evoked release of Ca_*i*_^2+^ was employed to validate the signaling profile of wild-type and novel 5-HT_2A_R and 5-HT_2C_R constructs. At 24 hours post transfection, HEK293 transiently expressing the WT 5-HT_2A_R, 5-HT_2A_R-CLuc, 5-HT_2A_R-RLuc, WT 5-HT_2C_R, 5-HT_2C_R-NLuc or 5-HT_2C_R-eYFP were washed with PBS, trypsinized (Gibco, Invitrogen, Waltham, MA), harvested, and plated in phenol-free DMEM with 10% dialyzed FBS at a density of 60,000 cells/well into poly-L-lysine coated (Sigma Aldrich, Waltham, MA) 96-well black wall, clear bottom cell culture plates (Greiner Bio-One, Monroe, NC). The next day (~18 hours later), complete media was aspirated and replaced with 50 μl of Hank’s balanced salt solution (HBSS) and cells serum starved for one hour in an incubator. In preparation for measurement of Ca_*i*_^2+^ release on a fluorescence imaging plate reader (FLIPR Tetra^®^; Molecular Devices, Sunnyvale, CA), 50 μl of 2X FLIPR^®^ Calcium 5 dye (Molecular Devices, Sunnyvale, CA) with 2.5 mM probenecid was added to the cells and plates were returned to the incubator for one hour. Serial dilutions of 5-HT (10^−12^ to 10^−5^ M) were prepared at 5X final concentration and transferred to a 96-well source plate. Cell and 5-HT plates were placed in a fluorescence imaging plate reader (FLIPR^TETRA^; Molecular Devices, Sunnyvale, CA). The FLIPR^TETRA^ was programmed to read baseline dye fluorescence for 10 sec followed by addition of 25 μl (5X) drug/well and read for an additional 180 sec (acquisition once/sec). The maximum fluorescence (ΔF) observed in each well during the first 40 seconds after 5-HT addition was determined using the FLIPR^TETRA^ ScreenWorks 4.0 program and results normalized to the average of the baseline fluorescence (F) in each well (first 10 reads); data are presented as the percentage of the WT 5-HT_2_ response. Data from four independent experiments, conducted in technical triplicate were analyzed.

### Luciferase complementation assay (LCA)

In the LCA reporter system, two complementary N- (NLuc) and C-terminus (CLuc) components of the enzyme luciferase, which have no activity on their own, are split in half and fused to the two receptor proteins of interest [33, 34, 40]. The detection of two proteins in close proximity in living cells is achieved as the association of the two proteins within < 50 nm brings the inactive luciferase fragments into close proximity and the luciferase enzyme activity is reconstituted [33, 34, 40]. At 24 hours post transfection, HEK293 cells co-expressing various ratios of 5-HT_2A_R-CLuc and 5-HT_2C_R-NLuc plasmids were washed with phosphate buffered saline (PBS), trypsinized (Gibco, Invitrogen, Waltham, MA), harvested, centrifuged (500 x g for four mins) and plated in phenol-free DMEM with 10% dialyzed FBS at a density of 60,000 cells/well into poly-L-lysine (Sigma Aldrich, Waltham, MA) coated white-walled clear bottom 96-well cell culture plates (Greiner Bio-One, Monroe, NC) and cultured overnight. Cells were serum starved for one hour in HBSS and luciferase activity was measured 48 hours post-transfection following the addition of 1.5 mg/mL *d*-luciferin (GoldBio, St Louis, MO) and 150 μM coenzyme A (Thermo Scientific, Waltham, MA). Total luminescence was measured with an H4 synergy reader (Biotek, Winooski, VT) for 40 mins following *d*-luciferin addition (time = -5 min). Maximal luminescence values from 45-min kinetic runs are plotted for comparison. Data from three independent experiments were analyzed and conducted in at least four technical replicates.

### Saturation binding assay

At 24 hours post-transfection, transfection media was replaced with DMEM supplemented with 10% dialyzed FBS; 24 hours later, cells were collected by centrifugation at 4000 x *g* at 4°C for 25 mins in ice cold assay buffer containing 50 mM Tris HCl, 10 mM MgCl_2_ and 0.1 mM EDTA. Membranes were collected by centrifugation three times at 4500 x g at 4°C for 20 mins and stored at -80 °C until use. Saturation binding isotherms were performed in 96-well plates using similar methods to the psychoactive drug screening program (PDSP) [41]. For saturation binding assays, 0.2 to 20 nM of [^3^H]-ketanserin (PerkinElmer, Waltham, MA) for 5-HT_2A_R or [^3^H]-mesulergine (PerkinElmer, Waltham, MA) for 5-HT_2C_R was used to obtain affinity (K_D_) and protein concentration (B_MAX_) values following co-transient transfection with the 5-HT_2C_R-NLuc and 5-HT_2A_R-CLuc receptor constructs into HEK293 cells. Non-specific binding was determined in the presence of 10 μM of mianserin hydrochloride (Sigma Aldrich, St. Louis MO). The reaction mixtures were incubated at room temperature (RT) for 90 mins on a plate shaker to reach equilibrium, and then passed rapidly through a printed filtermat using a FilterMate Harvester (PerkinElmer, Waltham, MA). The printed filtermat containing bound [^3^H]-ketanserin or [^3^H]-mesulergine was microwaved for one min to dry, then a MeltiLex sheet was melted onto the printed filtermat via a hotplate. The contents were sealed and counted for scintillation using a MicroBeta 2 (PerkinElmer, Waltham, MA). Direct radioligand concentrations were measured by pipetting into 1 mL of Optiphase Supramix (PerkinElmer, Waltham, MA) and measured on a Tri-Carb 2910TR liquid scintillation analyzer (PerkinElmer, Waltham, MA). Protein concentrations were determined using the bicinchoninic acid (BCA) protein assay kit (Thermo Scientific, Waltham, MA) by measuring absorbance values (562 nm) on a H4 synergy reader (Biotek, Winooski, VT). Each experiment was performed in technical triplicates with a minimum of three biological replicates.

### MTT assay

The 3-(4,5-dimethylthiazol-2-yl)-2,5-diphenyltetrazolium bromide (MTT) assay for measurement of metabolic activity, an indicator of cell viability and health, was performed, according to manufacturer’s protocols (Sigma Aldrich, St. Louis MO). At 24 hours post-transfection, HEK293 cells co-transiently transfected with 0.75 μg of 5-HT_2C_R-NLuc, 0.25 μg of 5-HT_2A_R-CLuc, and increasing concentrations (0–2 μg) of either WT 5-HT_2C_R, 5-HT_2A_R or empty pcDNA3.1+ vector were washed with PBS, trypsinized (Gibco, Invitrogen, Waltham, MA), harvested, and plated in DMEM with 10% dialyzed FBS at a density of 60,000 cells/well into poly-L-lysine coated (Sigma Aldrich, Waltham, MA) clear 96-well cell culture plates (Greiner Bio-One, Monroe, NC). Cells were serum starved for one hour in HBSS, or 500 μM of H_2_O_2_ (positive control), then 0.5 mg/ml MTT labeling reagent added for four hours at 37°C. Next, 100 μL of solubilization solution was added and incubated overnight at 37°C. The following day absorbance values at 550 nm and 590 nm were measured with an H4 synergy reader (Biotek, Winooski, VT). Each experiment was performed in technical triplicate with a minimum of three biological replicates.

### Proximity ligation assay (PLA)

The PLA is a flexible and informative technology that expands upon traditional immunocytochemistry to include direct detection of low levels of individual proteins, the existence of protein:protein interactions (≤ 40 nm) as well as the subcellular localization of the protein:protein interaction with high specificity and sensitivity. The PLA was performed using the recommended manufacturer’s protocol with minor modification, similar to those previously reported [42]. The 5-HT_2A+2C_R-CHO cells were plated in poly-D-lysine coated 16-well chamber slides (Thermo Scientific, Nunc Lab-Tek) in 100 μl growth media at the density of 16K cells/well and placed overnight in the incubator. The following day (~18 hours later), cells were fixed in 100 μl of freshly made, cold paraformaldehyde (PFA) in PBS (pH 7.4) for a final concentration of 4% PFA. After 15 min incubation at RT, the walls of the chamber slides were removed and slides washed twice with PBS. Next, cells were permeabilized for 10 min in 100 uM digitonin (Crescent Chemical Co., Islandia, NY) in PBS, washed twice in PBS with 0.1% Tween 20 (PBS-T) and blocked with 4% normal donkey serum (NDS) (Jackson ImmunoResearch Laboratories, West Grove, PA) in PBS-T. Cells were then incubated with rabbit anti-5-HT_2A_R (1:500; LS-C172270, LifeSpan Biosciences, Seattle, WA) and/or mouse anti-5-HT_2C_R (1:50; sc-17797, Santa Cruz Biotechnology, Dallas, TX) in 4% NDS in PBS-T and left overnight in 4°C. After washing the slides 5X with RT PBS-T, the samples were processed for the PLA according to manufacturer’s protocol with minor modifications (Sigma, Duolink^®^); cells were incubated with the appropriate combination of secondary antibodies conjugated with oligonucleotide probes for the PLA (Mouse PLUS; Mouse MINUS, Rabbit PLUS, Rabbit MINUS; Sigma, Duolink^®^ In Situ PLA Probes: DUO92001-2; DUO92004-5) in 4% NDS in PBS-T for one hour in an incubator. To remove unbound PLA probes, slides were washed twice in Duolink^®^ washing buffer A (Sigma, Duolink^®^ In Situ Wash Buffers, Fluorescence, DUO82049) at RT and incubated with Duolink^®^ ligation solution (Sigma, Duolink^®^, In Situ Detection Reagents Red; DUO92008) for 30 min in an incubator. Cells were then washed twice with RT Duolink^®^ washing buffer A and Duolink^®^ amplification-polymerase solution was applied to the slides. After 100 min incubation in the dark, slides were washed with Duolink^®^ washing buffer B (Sigma, Duolink^®^ In Situ Wash Buffers, Fluorescence, DUO82049) at RT followed by rinsing with 0.01x RT Duolink^®^ washing buffer B. On the dried slides, mounting media (Sigma, Duolink^®^ In situ Mounting Medium with DAPI, DUO082040) was applied and cells were imaged the same day with a Leica True Confocal Scanner SPE and Leica Application Suite Advanced software (Leica Microsystems, Wetzlar, Germany). Four non-overlapping fields of view per well (20X magnification) were identified and photomicrographs acquired under each experimental condition. Images were acquired with Leica LASX Software and processed with NIH ImageJ [43, 44]. To discriminate PLA puncta from the background fluorescence, identical for all conditions, the manually selected threshold (70) was applied to all images. The number of nuclei (DAPI+; ~170 per field of view per condition) and total puncta (red spots) were counted using the Duolink^®^ Image Tool Software (Olink Bioscience) from each of the four field of views and averaged for each experimental condition for statistical comparison, with a total of five biological replicates.

### Bioluminescence resonance energy transfer (BRET) assay

BRET is a biophysical technique that utilizes energy transfer between Renilla luciferase (RLuc) and a fluorescent protein, such as yellow fluorescent protein (YFP), that enables the spatial resolution of protein:protein interactions to ≤ 10 nm in living cells. The BRET assay is commonly used to characterize protein:protein interactions with GPCRs and was adapted from previous publications with minor modifications [8, 45]. At 24 hours post transfection, HEK293 cells co-expressing various ratios of 5-HT_2A_R-RLuc or β_2-_AR-Rluc and 5-HT_2C_R-eYFP plasmids were washed with PBS, trypsinized (Gibco, Invitrogen, Waltham, MA), harvested, centrifuged (500 x g for four mins), and plated in phenol-free DMEM with 10% dialyzed FBS at a density of 60,000 cells/well into poly-L-lysine (Sigma Aldrich, Waltham, MA) coated white-walled, clear bottom 96-well cell culture plates (Greiner Bio-One, Monroe, NC) and cultured overnight. The following day, cells were serum starved for one hour in HBSS and BRET activity was measured 48 hours post-transfection following the addition of 50 μM (5 μM final concentration) coelentrazine H (Fisher Scientific, Waltham, MA). Luminescence and fluorescence were measured simultaneously with an H4 synergy reader (Biotek, Winooski, VT) using 528/20 and 460/40 filters, respectively, 15 mins following coelentrazine H addition. The BRET ratio was calculated as [(emission at 528 nm/ emission at 460 nm)–(background at 528 nm/ background at 460 nm)]; background corresponds to the signal in cells expressing the RLuc fusion protein alone under similar conditions. For improved readability, the results were displayed in milli-BRET units (mBRET), with one mBRET corresponding to the BRET ratio multiplied by 1,000. Each experiment was performed in technical quadruplicates with a minimum of three biological replicates.

### Data analysis and statistics

All data were analyzed with GraphPad Prism 7.0 software (La Jolla, CA). Maximal luminescence values (± SEM) from the LCA and MTT assays were compared using a one-way analysis of variance (ANOVA) followed by *a priori* comparisons conducted with a Tukey’s test. We assessed the potency (pEC_50_) and efficacy (E_MAX_) of 5-HT to activate Ca_*i*_^2+^ release for all novel receptor fusion proteins to ensure similar signaling properties relative to the wildtype receptors. Data from Ca_*i*_^2+^ release assays are presented as half maximum (pEC_50_) and maximum (E_MAX_) values (mean ± SEM), representing potency and efficacy, respectively, as computed by GraphPad using a four parameter nonlinear regression curve-fitting algorithm. To assess the effect of different transfection ratios of receptor plasmids on the total receptor protein level, we compared B_max_ values obtained from radioligand binding studies. We also determined the potential effect of different transfection ratios of receptor plasmids on receptor ligand affinity, by statistically comparing receptor affinity values (K_d_). Data from saturation binding isotherms are presented as B_MAX_ and K_d_ values (mean ± SEM), as computed by GraphPad using one site-specific binding nonlinear regression curve-fitting algorithm; a one-way ANOVA was conducted followed by *a priori* comparisons with Dunnett’s test. The total number of PLA puncta (mean ± SEM) from four individual fields of view under each experimental condition were averaged for each separate condition; a one-way ANOVA followed by *a priori* comparisons with a Tukey’s test or a Student’s t-test was conducted, as appropriate. Data from BRET assays are presented as mBRET_50_ and mBRET_MAX_ values (mean ± SEM) as computed by GraphPad using specific binding with Hill slope nonlinear regression curve-fitting algorithm. The experiment wise error rate for all analyses was set at α = 0.05.

## Results

### Luciferase complementation analysis indicates 5-HT_2A_R:5-HT_2C_R interaction within 50 nm

**Fig 1:**
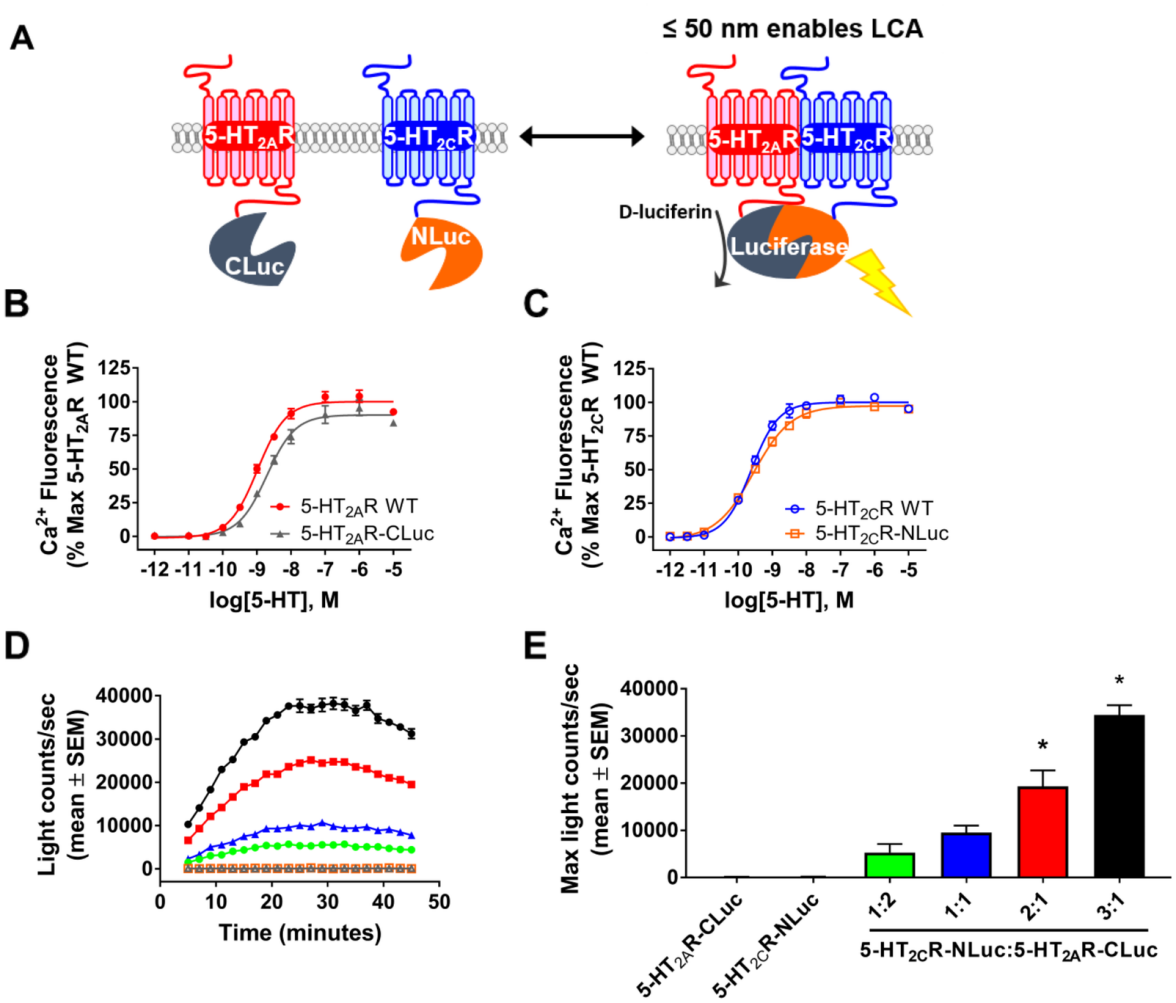
Luciferase complementation analysis indicates 5-HT_2A_R:5-HT_2C_R interaction within 50 nm. **(A)** Schematic of luciferase complementation assay (LCA) between 5-HT_2A_R-CLuc and 5-HT_2C_R-NLuc constructs (adapted from Luker et al., 2004) [33, 34]. **(B)** Representative concentration response curve for 5-HT-mediated intracellular calcium (Ca_*i*_^2+^) release for WT 5-HT_2A_R (red) and 5-HT_2A_R-CLuc (grey) constructs transiently transfected in HEK293 cells. The 5-HT-mediated increase in Ca_*i*_^2+^ dye fluorescence over baseline was calculated as the % maximal response WT 5-HT_2A_R. **(C)** Representative 5-HT concentration response curve for WT 5-HT_2C_R (blue) and 5-HT_2C_R-NLuc (orange) constructs transiently transfected in HEK293 cells. The 5-HT-mediated increase in Ca_*i*_^2+^ dye fluorescence over baseline was calculated as the % maximal response of WT 5-HT_2C_R. **(D)** Representative luminescence trace in HEK293 cells using varying transfection ratios (1:1, 2:1, 2:1, 3:1; 1 μg total plasmid DNA) of 5-HT_2C_R-NLuc and 5-HT_2A_R-CLuc constructs, respectively. Light counts/sec are shown at 1:2 (green), 1:1 (blue), 2:1 (red), and 3:1 (black) transfection ratios of 5-HT_2C_R-NLuc to 5-HT_2A_R-CLuc, respectively. **(E)** The maximal luminescence value from three independent experiments in which varying 5-HT_2C_R-NLuc:5-HT_2A_R-CLuc transfection ratios were tested. *p < 0.05 *vs*. 1:1 5-HT_2C_R-NLuc:5-HT_2A_R-CLuc transfection ratio.

We developed a split luciferase complementation assay (LCA) in which half of luciferase is attached to the C-terminus of each receptor (5-HT_2A_R-CLuc and 5-HT_2C_R-NLuc, respectively) to test if the 5-HT_2A_R and 5-HT_2C_R interact in a complex in living cells. If the association of the two receptors occurs in close proximity (< 50 nm), then a functional luciferase is reconstituted and enzymatic activity assessed by emitted luminescence (Fig 1A) [33, 34]. We created 5-HT_2A_R-CLuc and 5-HT_2C_R-NLuc split luciferase constructs and initially tested the functional signaling activity of the engineered receptors in transfected HEK293 cells. Both 5-HT_2A_R and 5-HT_2C_R are Gα_q/11_-coupled receptors which, upon activation, stimulate Ca_*i*_^2+^ release [18, 19]. Thus, concentration response curves (10^−12^ to 10^−5^) for 5-HT-mediated Ca_*i*_^2+^ release were generated to determine the functional activity of the wildtype and engineered receptors (Fig 1B and 1C). Serotonin-mediated Ca_*i*_^2+^ release was similar in cells transfected with the 5-HT_2A_R-CLuc (Fig 1B) or 5-HT_2C_R-NLuc (Fig 1C) relative to cells transfected with WT 5-HT_2A_R (Fig 1B) or WT 5-HT_2C_R (Fig 1C). The concentration of 5-HT required to induce calcium release (potency) and the magnitude of calcium released (efficacy) were both compared. The potency (pEC_50_ = 9.20 ± 0.27) and efficacy (E_MAX_ = 91 ± 8%) of 5-HT for the 5-HT_2A_R-CLuc were not significantly different than the potency (pEC_50_ = 9.07 ± 0.09) and efficacy (E_MAX_ = 100 ± 6%) of 5-HT for WT 5-HT_2A_R (*p* > 0.05; Fig 1B). Likewise, the potency (pEC_50_ = 9.41 ± 0.16) and efficacy (E_MAX_ = 97 ± 8%) of 5-HT for the 5-HT_2C_R-NLuc were not significantly different than the potency (pEC_50_ = 9.54 ± 0.13) and efficacy (E_MAX_ = 100 ± 8%) of 5-HT for the WT 5-HT_2C_R (*p* > 0.05; Fig 1C). Thus, these data demonstrate that the engineered 5-HT_2A_R-CLuc and 5-HT_2C_R-NLuc exhibit 5-HT-mediated signaling as seen for the native receptors when expressed in live cells.

We co-transfected HEK293 cells with 5-HT_2C_R-NLuc and 5-HT_2A_R-CLuc at various plasmid DNA ratios and assessed luciferase activity to determine if co-expression of 5-HT_2A_R-CLuc and 5-HT_2C_R-NLuc would reconstitute luciferase activity, which would indicate formation of a receptor complex. Bioluminescence light counts/sec are shown at 1:2 (green), 1:1 (blue), 2:1 (red), and 3:1 (black) transfection ratios of 5-HT_2C_R-NLuc to 5-HT_2A_R-CLuc plasmid DNA, respectively; luminescence was recorded once every four mins over a 45-min period (Fig 1D). The time course illustrates that robust luciferase complementation was observed at the 2:1 and 3:1 transfection ratios of 5-HT_2C_R-NLuc:5-HT_2A_R-CLuc (Fig 1D); no significant luminescence was detected following transfection of the negative controls 5-HT_2C_R-NLuc alone (orange) or 5-HT_2A_R-CLuc (grey) alone (Fig 1D). A main effect of transfection ratio on maximum light counts/sec was detected [F_5, 12_ = 49.79; p < 0.0001; Fig 1E]; the maximum light counts/sec was significantly elevated for the 2:1 and 3:1 transfection ratios of 5-HT_2C_R-NLuc:5-HT_2A_R-CLuc (*p* < 0.05 *vs*. 1:1 transfection ratio). To determine expressed receptor protein levels, we performed saturation radioligand binding for the 5-HT_2A_R (with [^3^H]-ketanserin) and 5-HT_2C_R (using [^3^H]-mesulergine) in membranes extracted from cells at 1:1, 2:1, and 3:1 transfection ratios of 5-HT_2C_R-NLuc:5-HT_2A_R-CLuc. A main effect of transfection ratio on total receptor level (B_MAX_) for the 5-HT_2C_R was detected [F_2,6_ = 5.67; *p* < 0.05; Table 1]; the 3:1 5-HT_2C_R-NLuc:5-HT_2A_R-CLuc transfection ratio yielded a significant increase in the B_MAX_ for 5-HT_2C_R *vs*. the 1:1 5-HT_2C_R-NLuc:5-HT_2A_R-CLuc transfection ratio (p < 0.05; Table 1). A main effect of transfection ratio on total receptor level (B_MAX_) for the 5-HT_2A_R was detected [F_2,6_ = 6.16; *p* < 0.05; Table 1]; the 3:1 5-HT_2C_R-NLuc:5-HT_2A_R-CLuc transfection ratio yielded a significant decrease in the B_MAX_ for 5-HT_2A_R *vs*. the 1:1 5-HT_2C_R-NLuc:5-HT_2A_R-CLuc transfection ratio (p < 0.05; Table 1). No main effect of 5-HT_2C_R-NLuc:5-HT_2A_R-CLuc transfection ratio on the ligand binding affinity (K_d_) for [^3^H]-ketanserin at the 5-HT_2A_R-CLuc [F_2,6_ = 0.07; p > 0.05] or the ligand binding affinity (K_d_) for [^3^H]-mesulergine at the 5-HT_2C_R-NLuc was detected [F_2,6_ = 0.24; *p* > 0.05]. Moreover, the 3:1 5-HT_2C_R-NLuc:5-HT_2A_R-CLuc transfection ratio resulted in a protein ratio of 0.408 (Table 1), indicating a molar excess of 5-HT_2A_R to 5-HT_2C_R protein. Ideally, a 1:1 protein ratio would be achieved for these studies, however, the 5-HT_2A_R-CLuc consistently expresses at a higher efficiency and level than the 5-HT_2C_R-NLuc in HEK293 cells, as observed in Table 1. Further conditions to attempt to achieve a 1:1 protein ratio (i.e., above the 3:1 plasmid ratio) negatively impacted the health of the cells using the MTT assay (data not shown). Thus, we were unable to obtain a higher expression of the 5-HT_2C_R without increasing the overall amount of transfected plasmid DNA and negatively impacting cell health. Taken together, these data suggest that a complex formation exists within 50 nm (limit of detection for the LCA) and that complex formation increases proportionally to the 5-HT_2C_R:5-HT_2A_R expression ratio.

**Table 1:** Protein levels and affinity values ascertained in HEK293 cells transiently co-transfected with the 5-HT_2C_R-NLuc and 5-HT_2A_R-CLuc at various ratios.

Transfection Ratio^a^	5-HT_2C_R-NLuc B_MAX_ (fmol/mg)^b^	[^3^H]-Mesulergine Kd (nM)^b^	5-HT_2A_R-CLuc B_MAX_ (fmol/mg)^b^	[^3^H]-Ketanserin Kd (nM)^b^	Protein Ratio^c^
1:1	451 ± 48	2.5 ± 0.3	2512 ± 224	1.8 ± 0.1	0.180
2:1	588 ± 55	2.3 ± 0.2	1887 ± 121	1.9 ± 0.3	0.312
3:1	692 ± 49*	2.2 ± 0.4	1697 ± 154*	1.8 ± 0.2	0.408

^*a*^ Different ratios of vectors (1:1, 2:1, 3:1) of 5-HT_2C_R-NLuc and 5-HT_2A_R-CLuc were co-transiently transfected into HEK293 cells and membranes were isolated.

^*b*^ B_MAX_ (fmol/mg protein) and K_d_ (nM) values (mean ± SEM) were determined for the 5-HT_2C_R-NLuc ([^3^H]-mesulergine) and 5-HT_2A_R-CLuc ([^3^H]-ketanserin).

^*c*^ The protein ratio was calculated as the B_MAX_ for 5-HT_2C_R-NLuc to the B_MAX_ for 5-HT_2A_R-CLuc.

* *p* < 0.05 *vs*. 1:1 5-HT_2C_R-NLuc:5-HT_2A_R-CLuc transfection ratio

**Fig 2:**
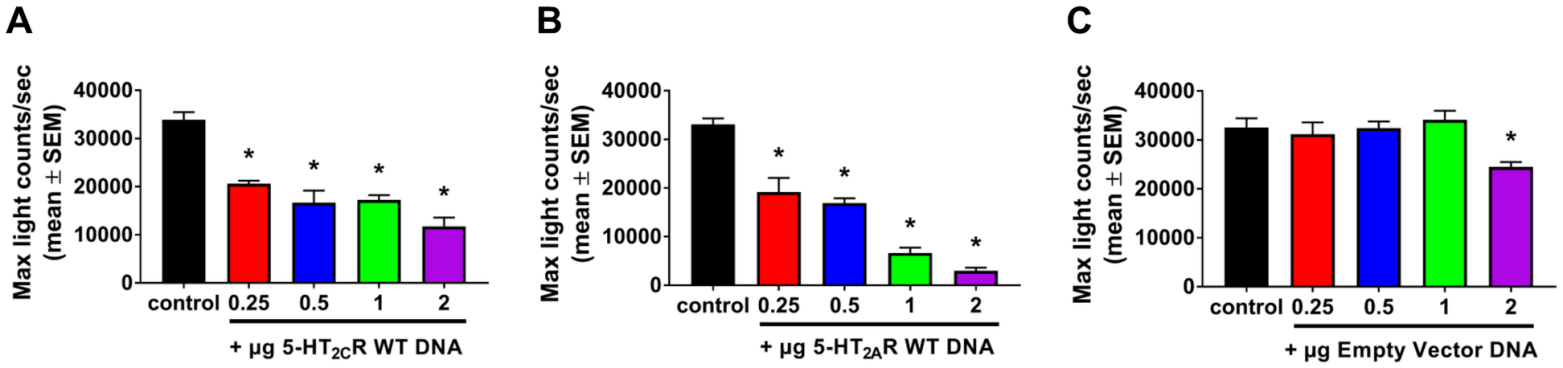
Wildtype receptor attenuates the 5-HT_2A_R:5-HT_2C_R luciferase complementation. The maximal luminescence value for the 5-HT_2C_R-NLuc:5-HT_2A_R-CLuc 3:1 transfection ratio (control) in the presence of varying amounts (0.25–2 μg) of **(A)** WT 5-HT_2C_R, **(B)** WT 5-HT_2A_R, or **(C)** empty vector. All results are maximal luminescence values from four independent experiments. *p < 0.05 *vs*. control.

We next determined if the LCA interaction could be inhibited by co-transfection of the 5-HT_2C_R-NLuc:5-HT_2A_R-CLuc (3:1 transfection ratio, Fig 2, “control”) with increasing amounts of cDNA (0.25–2 μg) for untagged WT 5-HT_2C_R (Fig 2A), WT 5-HT_2A_R (Fig 2B) or empty vector (Fig 2C). We predicted that transfection of the untagged WT 5-HT_2_R would competitively interact with its respective 5-HT_2_R LCA construct for the heteromeric complex, thereby reducing the ability to reconstitute luciferase, resulting in a reduction in the luminescence signal. A main effect of the WT 5-HT_2C_R transfection condition on maximum light counts/sec was detected [F_4, 12_ = 21.13; p < 0.0001; Fig 2A]; the native WT 5-HT_2C_R at all amounts of cDNA co-transfected decreased the luminescence (mean ± SEM) of the 5-HT_2C_R-NLuc:5-HT_2A_R-CLuc (*p* < 0.05 *vs*. control; Fig 2A). A main effect of the WT 5-HT_2A_R transfection condition on maximum light counts/sec was detected [F_4, 12_ = 68.83; p < 0.0001; Fig 2B]; the native WT 5-HT_2A_R at all amounts of cDNA co-transfected decreased the luminescence of the 5-HT_2C_R-NLuc:5-HT_2A_R-CLuc (*p* < 0.05 *vs*. control; Fig 2B) A main effect of the empty vector transfection condition on maximum light counts/sec was detected [F_4, 11_ = 4.034; *p* < 0.05; Fig 2C]; the empty vector at 2 μg decreased the luminescence of the 5-HT_2C_R-NLuc:5-HT_2A_R-CLuc (*p* < 0.05 *vs*. control; Fig 2C). Taken together, these data indicate that the luciferase complementation between the 5-HT_2A_R:5-HT_2C_R is not due to a random complementation of the luciferase fragments.

We next employed an MTT cell viability assay to determine if cellular metabolic activity was affected following these various plasmid co-transfections (S1 Fig). A one-way ANOVA indicated a main effect of experimental condition on absorbance (F_13,28_ = 12.34; *p* < 0.0001); the positive control H_2_O_2_, which increases reactive oxygen species causing cell death, significantly suppressed absorbance, indicating a decrease in cell viability (S1A Fig). No effect of WT 5-HT_2C_R (S1A Fig), WT 5-HT_2A_R (S1B Fig) or empty vector (S1C Fig) on absorbance was detected (*p* > 0.05 *vs*. control), suggesting that DNA overexpression, at the levels in which inhibition of the LCA signal was observed, did not negatively affect cell viability.

### Proximity ligation analysis indicates WT 5-HT_2A_R:5-HT_2C_R interaction within 40 nm

**Fig 3:**
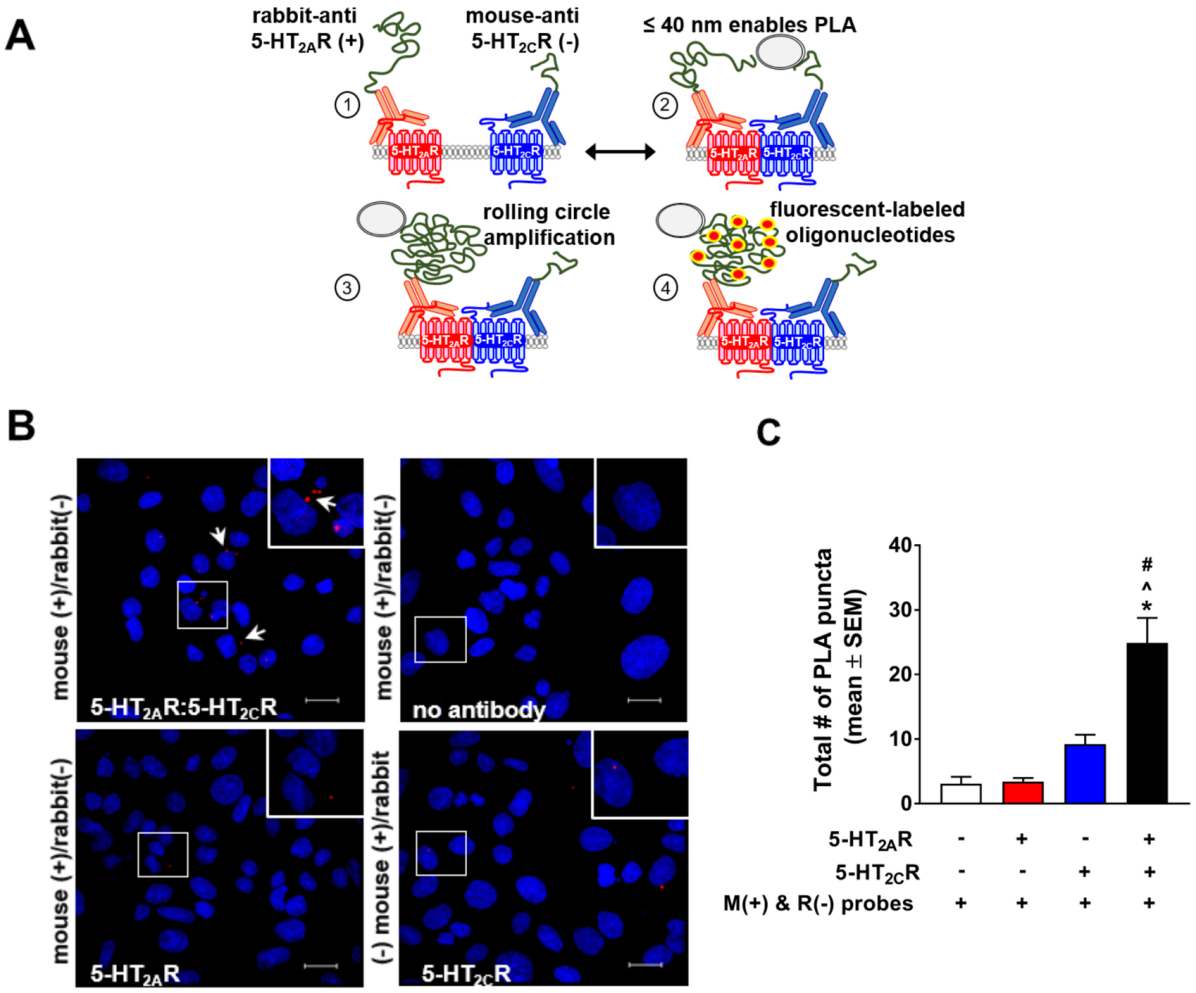
Proximity ligation assay indicates 5-HT_2A_R:5-HT_2C_R interaction within 50 nm. **(A)** Schematic of proximity ligation assay (PLA) between 5-HT_2C_R and 5-HT_2A_R. **(B)** Representative 60X confocal photomicrographs of PLA signal from 5-HT_2C_R and 5-HT_2A_R heteromeric formation (red puncta) and associated negative controls. PLA was performed using 5-HT_2A_R (rabbit polyclonal) and 5-HT_2C_R (mouse monoclonal) primary antibody and oligonucleotide-linked PLA secondary probes [mouse, M(+) and rabbit, R(-)]. Scale bars represent 10 μm. **(C)** Quantification of puncta from 20X photomicrographs from five independent experiments. *p < 0.05 *vs*. M(+) and R(-) probes only; ^*p* < 0.05 *vs*. 5-HT_2A_R plus M(+) and R(-) probes; #*p* < 0.05 *vs*. 5-HT_2C_R plus M(+) and R(-) probes.

We tested the hypothesis that the 5-HT_2A_R and 5-HT_2C_R interact using a proximity ligation assay (PLA) conducted in a cell line stably expressing both receptors (5-HT_2A+2C_R-CHO cells). In the PLA, when proteins of interest are in complex within close spatial proximity (≤ 40 nm), short DNA strands complement, ligate and allow rolling circle amplification to produce fluorescent signals [28, 42, 46, 47] (Fig 3A). The PLA was employed to identify and confirm expression of the 5-HT_2A_R (S2A Fig**, top left**) and 5-HT_2C_R (S2A Fig, **top right**) protomers in the dual-expressing cell line. Reverse transcription of RNA followed by qRT-PCR confirmed that 5-HT_2A+2C_R-CHO cells expressed both 5-HT_2A_R mRNA (ΔC_t_ = 7.67 ± 0.10) and 5-HT_2C_R mRNA (ΔC_t_ = 14.18 ± 0.13), but did not express 5-HT_2B_R mRNA (crossing threshold not determined). Similar to the transiently transfected HEK293 cells employed herein, the 5-HT_2A_R transcript was in greater abundance relative to the 5-HT_2C_R transcript in the stably expressing 5-HT_2A+2C_R-CHO cells. Experimental controls in which the appropriate primary antibody was omitted and the PLA probe included (e.g., rabbit+/rabbit-; mouse+/mouse-) demonstrated no puncta, as expected (S2A Fig, **bottom**). The total number of puncta specific to 5-HT_2A_R (*p* < 0.05; S2B Fig) or 5-HT_2C_R (*p* < 0.05; S2B Fig) was significantly higher relative to controls. We then quantified 5-HT_2A_R and 5-HT_2C_R co-localized in the native environment of the dual expressing 5-HT_2A+2C_R-CHO cells. We observed a distinct positive signal (red puncta spot) indicating that the 5-HT_2A_R and 5-HT_2C_R are in close proximity in the dual expressing cell line (Fig 3B, **top**). To provide sufficient controls and to ensure that observed fluorescent signal was caused by a receptor:receptor interaction, and not due to nonspecific binding of the antibodies, cells in separate wells were labeled no primary antibody, the anti-5-HT_2A_R antibody or the anti-5-HT_2C_R antibody alone (Fig 3B). A main effect of experimental conditions for the total number of puncta in the 5-HT_2A+2C_R-CHO cells was detected [F(3, 16) = 22,59; *p* < 0.05; Fig 3C]; the total number of puncta was significantly higher *vs*. all provided control conditions (*p* < 0.05; Fig 3C), indicating specificity of the antibody labeling. These data support the conclusion that WT 5-HT_2A_R and 5-HT_2C_R interact in cells within 40 nm of each other (limit of detection for PLA) [28, 42, 46, 47].

### BRET analyses indicates 5-HT_2A_R:5-HT_2C_R interaction within 10 nm

**Fig 4:**
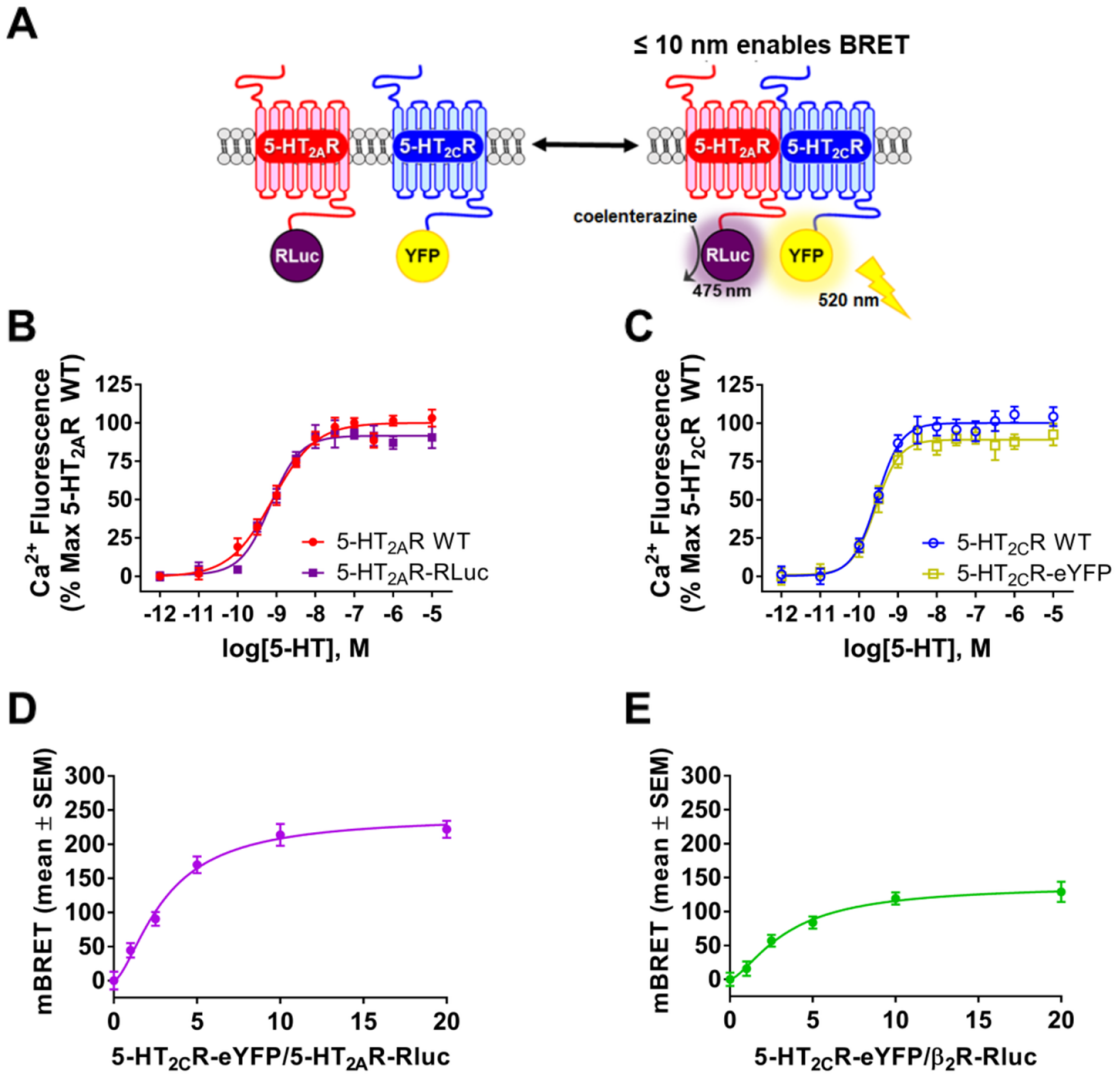
BRET indicates 5-HT_2A_R:5-HT_2C_R interaction within 10 nm. **(A)** Schematic of BRET assay between 5-HT_2C_R-RLuc and 5-HT_2A_R-eYFP constructs. **(B)** Representative concentration response curve for 5-HT-mediated intracellular calcium (Ca_*i*_^2+^) release for WT 5-HT_2A_R (red) and 5-HT_2A_R-RLuc (purple) constructs transiently transfected in HEK293 cells. The 5-HT-mediated increase in Ca_*i*_^2+^ dye fluorescence over baseline was calculated as the % maximal response of WT 5-HT_2A_R. **(C)** Representative concentration response curve for 5-HT-mediated Ca_*i*_^2+^ release for WT 5-HT_2C_R (blue) and 5-HT_2C_R-eYFP (yellow) constructs transiently transfected in HEK293 cells. The 5-HT-mediated increase in Ca_*i*_^2+^ dye fluorescence over baseline was calculated as the % maximal response of WT 5-HT_2A_R. **(D)** Representative BRET curve from HEK293 cells co-transfected with constant amount 5-HT_2A_R-RLuc (100 ng; donor construct) and increasing amounts of the 5-HT_2C_R-eYFP (1–20 fold excess; acceptor construct). **(E)** Representative BRET curve from HEK293 cells co-transfected with constant amount β_2_-AR-Rluc (100 ng; donor construct) and increasing amounts of the 5-HT_2C_R-eYFP (1–20 fold excess; acceptor construct). The mBRET signal was determined by calculating the ratio of light emitted at 528 nm to 460 nm (X 1000). Results obtained from four independent experiments.

To further resolve the spatial proximity of the receptors in live cells, we generated 5-HT_2A_R and 5-HT_2C_R constructs (donor: 5-HT_2A_R-RLuc; acceptor: 5-HT_2C_R-eYFP) for use in a BRET assay which requires interacting proteins be within 10 nm to generate resonance energy transfer (Fig 4A) [8]. To validate functionality of the engineered receptors, concentration response curves (10^−12^ to 10^−5^) for 5-HT-mediated Ca_*i*_^2+^ release were generated (Fig 4B and 4C). Serotonin-mediated Ca_*i*_^2+^ release was similar in cells transfected with the 5-HT_2A_R-RLuc (Fig 4B) or 5-HT_2C_R-eYFP (Fig 4C) relative to cells transfected with WT 5-HT_2A_R (Fig 4B) or WT 5-HT_2C_R (Fig 4C), respectively. The potency (pEC_50_ = 9.16 ± 0.08) and efficacy (E_MAX_ = 94 ± 7%) of 5-HT for the 5-HT_2A_R-RLuc were not significantly different than the potency (pEC_50 =_ 9.10 ± 0.05) and efficacy (E_MAX_ = 100 ± 6%) of 5-HT for WT 5-HT_2A_R (*p* > 0.05; Fig 4B). Likewise, the potency (pEC_50_ 9.57 ± 0.06) and efficacy (E_MAX_ = 90 ± 9%) of 5-HT for the 5-HT_2C_R-eYFP were not significantly different than the potency (pEC_50_ = 9.58 ± 0.08) and efficacy (E_MAX_ = 100 ± 5%) of 5-HT for the WT 5-HT_2C_R (*p* > 0.05; Fig 4C). Thus, these data demonstrate that the engineered 5-HT_2A_R-RLuc and 5-HT_2C_R-eYFP exhibit 5-HT-mediated signaling as seen for the native receptors when expressed in live cells.

A saturable BRET curve (mBRET_50_ = 3.0 ± 0.1; mBRET_MAX_ = 231 ± 8 mBRET units) in cells expressing a constant amount of donor construct (100 ng of 5-HT_2A_R-RLuc) and increasing amounts acceptor construct (1–20 fold of 5-HT_2C_R-eYFP) was obtained (Fig 4D), indicating a specific and saturable receptor:receptor interaction. To further evaluate the specificity of this interaction, we used the β_2_-AR-RLuc as a donor construct control as β_2_-AR-RLuc minimally associates with the 5-HT_2C_R-eYFP [48]. A lower, more linear BRET saturation curve (mBRET_50_ = 3.4 ± 0.1; mBRET_MAX_ = 135 ± 4 mBRET units) was observed in cells expressing a constant amount of donor construct (100 ng of β_2_-AR-RLuc) and increasing amounts of acceptor construct (1–20 fold of 5-HT_2C_R-eYFP) (Fig 4E). These results indicate the 5-HT_2A_R and 5-HT_2C_R form a close interaction within 10 nm of each other (limit of detection in BRET) in intact live cells.

## Discussion

The present study used a tiered strategy employing three complementary biophysical techniques with increasing spatial resolution to confirm that the 5-HT_2A_R and 5-HT_2C_R form a cellular level protein-protein complex interaction. In the LCA assay, we demonstrated that formation of the 5-HT_2A_R:5-HT_2C_R complex exists within 50 nm and increases proportionally to the 5-HT_2C_R:5-HT_2A_R protein expression ratio. Using the PLA, we found that cells stably expressing both the 5-HT_2A_R and 5-HT_2C_R exhibit 5-HT_2A_R:5-HT_2C_R heteroreceptor complexes within 40 nm of each other. Lastly, BRET analyses signify a specific and saturable 5-HT_2A_R:5-HT_2C_R interaction, indicating that the 5-HT_2A_R and 5-HT_2C_R form a close interaction within 10 nm of each other in intact live cells. Importantly, the bioengineered receptors generated for the LCA and the BRET exhibit 5-HT-mediated intracellular calcium signaling as seen for the native receptors, assuring that the tagged constructs do not impede receptor function. Thus, these studies support the conclusion that a very close 5-HT_2A_R:5-HT_2C_R heteromeric receptor interaction occurs in cultured cells.

Energy transfer-based technologies, such as BRET, have been fundamental in the development of a database of knowledge concerning the homo- and heterodimerization of GPCRs [45, 49, 50]. Considerable research has implicated that the energy transfer from the donor to the acceptor is below 10 nm [45, 49, 50]. We demonstrated a specific BRET saturation curve in cells expressing a constant amount of the 5-HT_2A_R-RLuc donor construct and increasing amounts of the 5-HT_2C_R-eYFP acceptor construct. A lower, more linear BRET saturation curve was seen in cells expressing a constant amount of donor β_2_-AR-RLuc construct and increasing amounts of acceptor 5-HT_2C_R-eYFP construct, a receptor pair previously suggested to minimally associate in live cells [48]. Because the diameter of the seven transmembrane helical core is estimated at ~5 nm, the observed, positive BRET signal strongly suggests that oligomerization has occurred between the 5-HT_2A_R and 5-HT_2C_R [45, 49, 50]. Furthermore, a reciprocal interaction between these two receptors is corroborated by previous analyses of molecular and pharmacological properties of the 5-HT_2A_R:5-HT_2C_R heterocomplex *in vitro* [8]. Intriguingly, this formation of the 5-HT_2A_R:5-HT_2C_R complex did not modify the Gα_q/11_ coupling of the protomers, but rather the 5-HT_2C_R exerts dominance when in complex with the 5-HT_2A_R, such that only the 5-HT_2C_R couples with the G protein to generate intracellular signaling; the 5-HT_2A_R signaling is ‘masked’ [8]. Thus, the 5-HT_2A_R:5-HT_2C_R protein complex appears to be a distinct molecular species that contributes to the control of cellular signaling, triggering unique intracellular signaling properties when co-expressed *in vitro* [8].

The detection of a 5-HT_2A_R:5-HT_2C_R protein complex in transfected cells [present results, [8] and rodent brain [5] extends observations of the co-localization of these receptors in the single neurons of the rat mPFC [15–17]. Our previous studies indicate that the lowest expression levels of the 5-HT_2A_R:5-HT_2C_R complex assessed by co-immunoprecipitation in the mPFC associates with the highest level of phenotypic impulsive action in the rat and that the ratio of 5-HT_2A_R to 5-HT_2C_R protein expression in the rat mPFC predicts the inherent level of motor impulsivity in individual rats [5]. Engineered 5-HT_2C_R knockdown in mPFC resulted in increased motor impulsivity [5, 51] concomitant with elevated 5-HT_2A_R expression and greater potency of the 5-HT_2A_R antagonist M100907 to suppress motor impulsivity [5]. Intriguingly, rats exposed to a low-protein diet *in utero* exhibited reduced 5-HT_2C_R and elevated 5-HT_2A_R expression in the hypothalamus concomitant with impaired sensitivity to 5-HT-mediated appetite in adulthood [52]. Although not yet linked to the formation or action of heteromers, these previous studies support a potential role for the 5-HT_2A_R:5-HT_2C_R interaction *in vivo* [11, 12].

The oligomerization of GPCRs has emerged as a vital property of receptor structure and function with identified macromolecular complexes comprising of at least two different protomers with biochemical properties that are discernibly distinct from those of the individual protomers [8, 45, 53]. The main functional GPCR unit for the 5-HT_2A_R [20, 21] and 5-HT_2C_R [22–25] is proposed to be their homomeric form, and recent studies suggest that different interacting homomers may constitute heteromeric complexes [53]. Future studies are needed to clarify the degree to which downstream signaling pathways are recruited by the individual receptors vs. heteroreceptor complexes, especially in terms of the level of ligand-directed signaling [54] as well as the functional effects of the 5-HT_2_R and their underlying role in behavior. Furthermore, drug discovery efforts selectively targeting either receptor should take into account the formation of a heteromeric complex when analyzing physiological responses [8].

## Supporting information

S1 Fig MTT assay for cell viability. The absorbance value for the 5-HT_2C_R-NLuc:5-HT_2A_R-CLuc 3:1 transfection ratio (control) in the presence of varying amounts (0.25–2 μg) of **(A)** WT 5-HT_2C_R, **(B)** WT 5-HT_2A_R, or **(C)** empty vector. All results are absorbance at 550 nm minus absorbance at 590 nm from four independent experiments * *p* < 0.05 *vs*. control.
(TIF)

S2 Fig Proximity ligation assay (PLA) for single receptor recognition. **(A)** Representative 60X confocal photomicrographs of PLA signal from 5-HT_2A_R (top left) and 5-HT_2C_R (top right) (red puncta) and associated negative controls (bottom). PLA was performed using 5-HT_2A_R (rabbit polyclonal) alone or 5-HT_2C_R (mouse monoclonal) primary antibody alone plus oligonucleotide-linked PLA secondary probes [rabbit, R(±); mouse, M(±)]. Scale bars represent 10 μm. **(B)** Quantification of puncta from 20X photomicrographs from five independent experiments. *p < 0.05 *vs*. M(±) probes only; ^*p* < 0.05 *vs*. R (±) probes only.
(TIF)

## Abbreviations

5-HT: serotonin
5-HT_2A_R: serotonin 5-HT_2A_ receptor
5-HT_2C_R: serotonin 5-HT_2C_ receptor
ANOVA: analysis of variance
β_2_-AR: β_2_-adrenergic receptor
B_MAX_: protein concentration
BRET: bioluminescence resonance energy transfer
CHO: Chinese hamster ovary cells
CLuc: C-terminal luciferase fragment
DMEM: Dulbecco’s modified Eagle’s medium
DMSO: dimethyl sulfoxide
eYFP: enhanced yellow florescent protein
FBS: fetal bovine serum
FKBP: human FK506-binding protein 12
FLIPR: fluorescence imaging plate reader
FRB: rapamycin-binding domain of human mammalian target of rapamycin
GPCR: G protein-coupled receptor
GHS1αR: growth hormone secretagogue receptor 1α
HBSS: Hank’s balanced salt solution
K_d_: affinity
mBRET: milli-BRET units
mPFC: medial prefrontal cortex
HEK293: human embryonic kidney cells
LCA: luciferase complementation assay
MTT: 3-(4,5-dimethylthiazol-2-yl)-2,5-diphenyltetrazolium bromide
NDS: normal donkey serum
NLuc: N-terminal luciferase fragment
PBS: phosphate buffered saline
PBS-T: PBS with 0.1% Tween 20
PLA: proximity ligation assay
PDSP: Psychoactive Drug Screening Program
pEC_50_: potency
RLuc: *Renilla* luciferase
RT: room temperature
