# Supplementary figures and images for: Biophysical validation of serotonin 5-HT2A and 5-HT2C receptor interaction
Source: PLoS One. 2018 Aug 29;13(8):e0203137. doi: 10.1371/journal.pone.0203137 (PMC6114921; doi:10.1371/journal.pone.0203137)

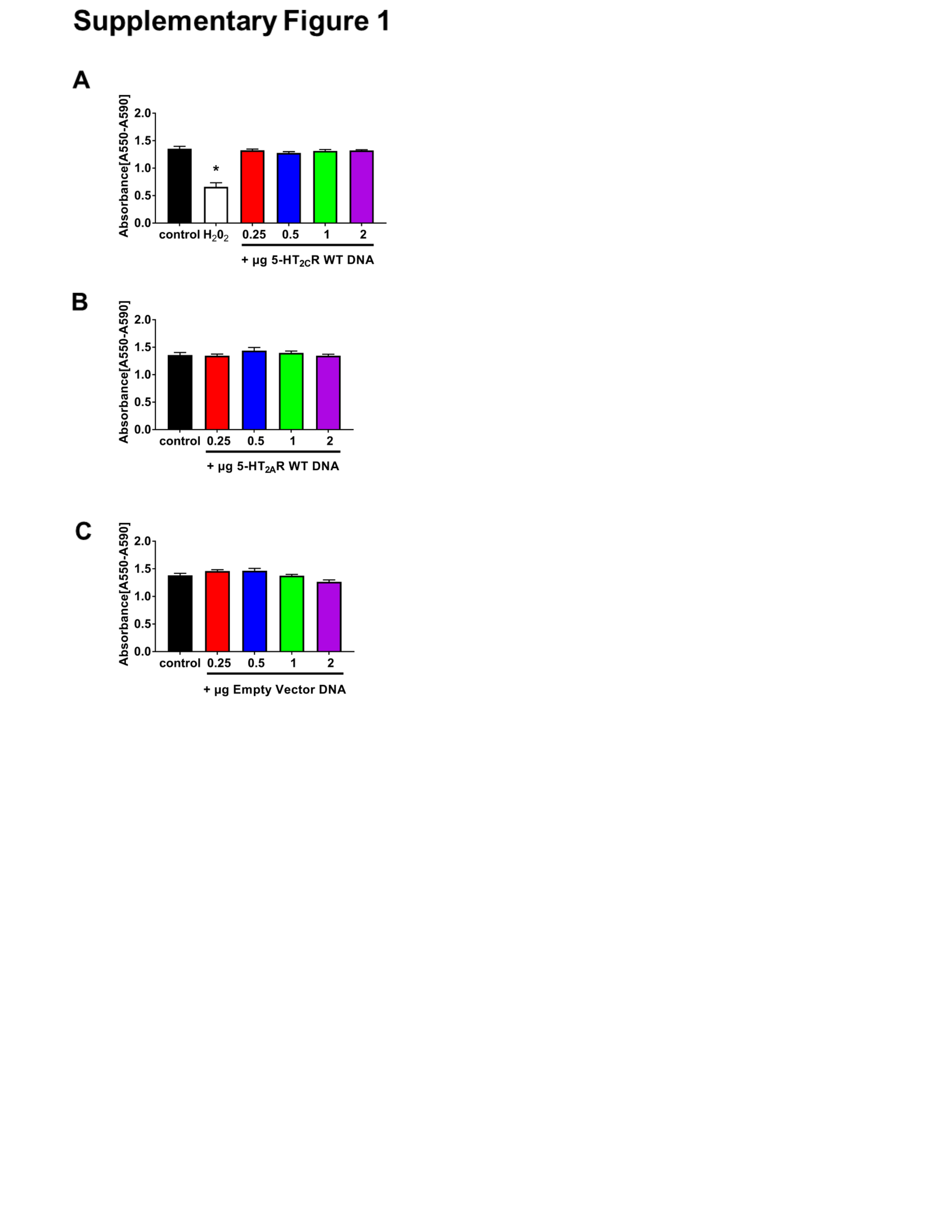

Supplement: S1 Fig — The absorbance value for the 5-HT2CR-NLuc:5-HT2AR-CLuc 3:1 transfection ratio (control) in the presence of varying amounts (0.25–2 μg) of (A) WT 5-HT2CR, (B) WT 5-HT2AR, or (C) empty vector. All results are absorbance at 550 nm minus absorbance at 590 nm from four independent experiments * p < 0.05 vs. control. (TIF) [file pone.0203137.s001.tif]

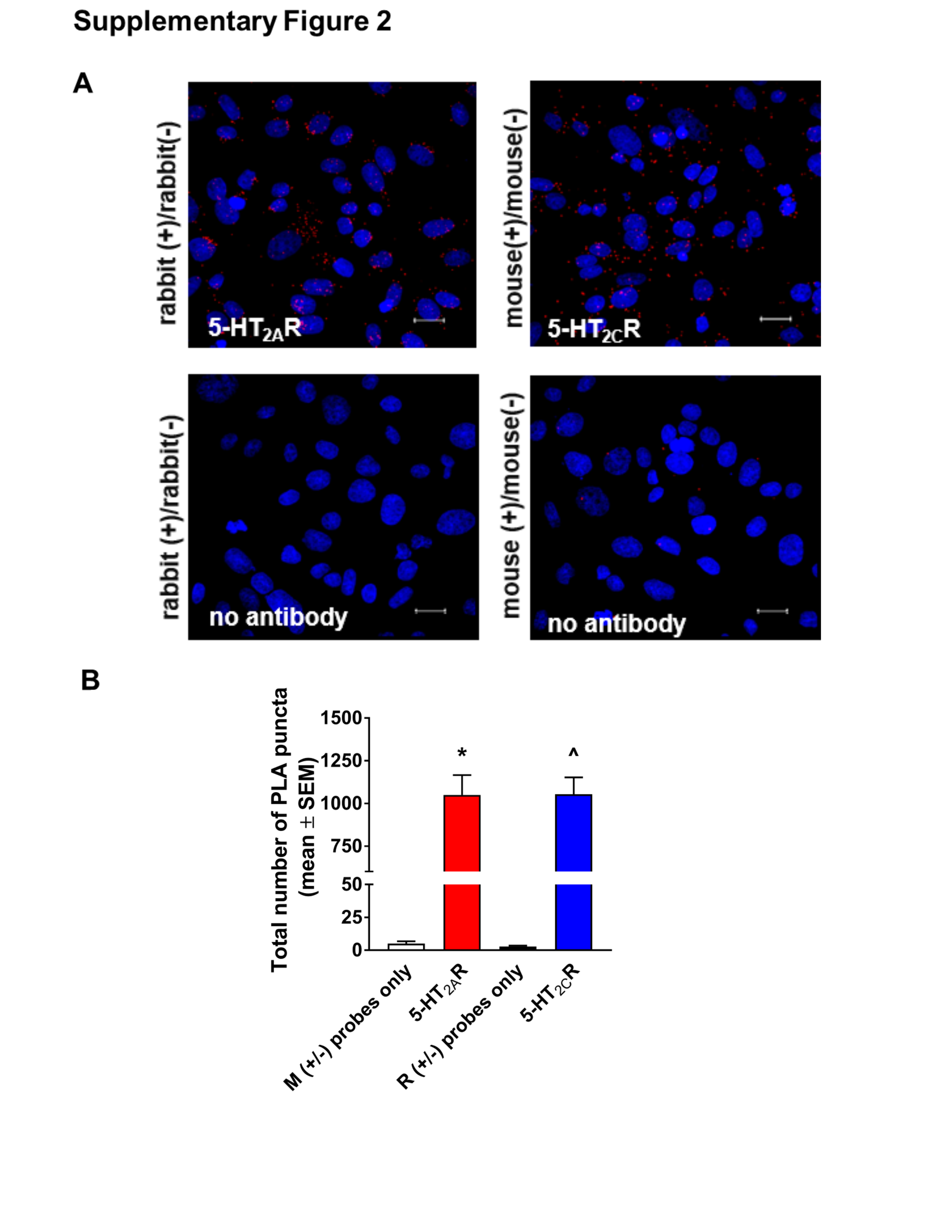

Supplement: S2 Fig — (A) Representative 60X confocal photomicrographs of PLA signal from 5-HT2AR (top left) and 5-HT2CR (top right) (red puncta) and associated negative controls (bottom). PLA was performed using 5-HT2AR (rabbit polyclonal) alone or 5-HT2CR (mouse monoclonal) primary antibody alone plus oligonucleotide-linked PLA secondary probes [rabbit, R(±); mouse, M(±)]. Scale bars represent 10 μm. (B) Quantification of puncta from 20X photomicrographs from five independent experiments. *p < 0.05 vs. M(±) probes only; ^p < 0.05 vs. R (±) probes only. (TIF) [file pone.0203137.s002.tif]
